# Cellular Immunity of Patients with Tuberculosis Combined with Diabetes

**DOI:** 10.1155/2022/6837745

**Published:** 2022-06-01

**Authors:** Peng Cheng, Liang Wang, Wenping Gong

**Affiliations:** ^1^Tuberculosis Prevention and Control Key Laboratory/Beijing Key Laboratory of New Techniques of Tuberculosis Diagnosis and Treatment, Senior Department of Tuberculosis, The 8th Medical Center of PLA General Hospital, Beijing 100091, China; ^2^Hebei North University, Zhangjiakou, 075000 Hebei, China; ^3^Department of Geriatrics, The 8th Medical Center of PLA General Hospital, Beijing 100091, China

## Abstract

Tuberculosis (TB) is one of humanity's three major infectious diseases. Diabetes mellitus (DM) is a metabolic disease characterized by hyperglycemia due to impaired insulin secretion or impaired insulin function. It has been reported that DM is a primary risk factor for TB disease. Given the increasing public health threat to people's health, more and more studies have focused on diabetes complicated by TB. Hyperglycemia can affect the function of human immune cells, promote primary infections and reactivation of TB, and increase the susceptibility and severity of TB. However, the immunological mechanism behind it is still not clear. By reviewing the related articles on tuberculosis complicated with diabetes published in recent years, this paper expounds on the effect of hyperglycemia on innate immunity and adaptive immunity of patients with TB. This review provides new insights for elucidating the immunological mechanism of TB complicated with DM and lays the foundation for finding potential targets for preventing and treating TB combined with DM.

## 1. Introduction

Tuberculosis (TB) is an infectious disease caused by *Mycobacterium tuberculosis* (MTB). According to the 2021 global tuberculosis report released by the World Health Organization (WHO), there were about 10 million new cases of TB and 1.3 million deaths in 2020 [1]. In addition, under the influence of the COVID-19 outbreak in 2019, the number of TB deaths rose by 100,000, bringing global efforts to reduce TB mortality back to the 2017 level continuously [1]. However, these data indicate that global TB prevention and control are still not optimistic.

Diabetes mellitus (DM) is an endocrine and metabolic disease caused by increased blood glucose levels due to insufficient insulin secretion or increased insulin resistance. Epidemiological studies show that in the past 30 years, the incidence of DM has risen from 3.5% to 5.0% in women and from 3.9% to 6.0% in men [2]. At present, about 80% of DM occurs in developing countries, and the survey found that the Middle East, North Africa, and South Asia had the highest percentage of increases and have become critical areas of DM prevalence. India and China are not only the most populous countries in the world but also the largest developing countries, and the numbers of patients with DM in the two countries are increasing. It is estimated that 116.4 million and 77 million people were affected by DM in both countries in 2019, respectively [2]. According to the International Diabetes Federation (IDF) report, the number of patients with DM worldwide is as high as 463 million in 2019 [3]. In other words, one out of ten people is suffering from DM.

As an independent risk factor for TB, DM has been confirmed in more than 40 studies to increase TB susceptibility. A meta-analysis shows that patients with DM are about three times more likely to develop TB than nondiabetic patients in low-and middle-income countries [4], and roughly the same results have been found in developed countries [5]. Furthermore, DM is related to aggravating the severity of TB infection, and it also has harmful effects on the clinical manifestation and treatment of TB [6]. Therefore, the dual burden of DM and TB has become a significant public health problem.

Previous studies have shown that in the TB patients complicated with DM (TB-DM), hyperglycemia and insulin resistance affect the function of innate and adaptive immune cells of the host, resulting in a decline in the ability of immunity to remove and kill MTB and an increase in mortality [7]. Compared to other recent review papers related to TB-DM, this review systematically explores the effects of DM on innate immunity and adaptive immunity of TB patients and highlights the potential immunological targets for the prevention, diagnosis, and treatment of TB-DM.

## 2. Characteristics of the Immune System in Patients with TB-DM

**Figure 1:**
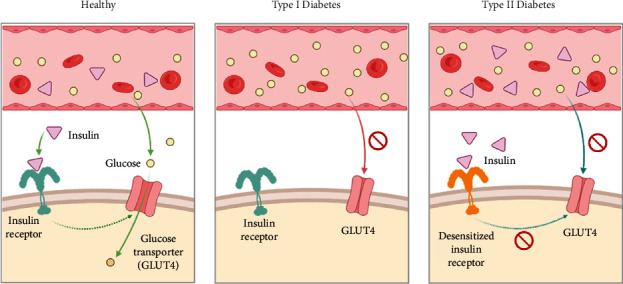
Pathogenesis of T1DM and T2DM. In healthy people, insulin binds to insulin receptors and activates the opening of glucose transporter channels in fat/muscle cells, thereby reducing blood glucose levels. In T1DM, pancreatic cells are unable to produce insulin and therefore cannot bind to insulin receptors to induce glucose transporters to remove glucose from the blood. In people with T2DM, chronic overproduction of insulin leads to desensitization of insulin receptors so that glucose transporters cannot be activated to remove glucose from the blood.

DM is chronic hyperglycemia caused by insufficient insulin secretion or increased insulin resistance in the body [8], including type I (T1DM) and type II (T2DM) diabetes (Figure 1). Previous studies have found that chronic hyperglycemia in DM can cause a series of immunological changes, including (1) inflammation induces a decrease in dendritic cells (DCs) [9]; (2) increased macrophage infiltration leads to increased secretion of proinflammatory factors, which will result in islet *β*-cell dysfunction and apoptosis [10]; (3) higher levels of reactive oxygen species (ROS) produced by neutrophils increase the risk of organ injury [11]; (4) the number of natural killing (NK) cells increases but they are dysfunctional characterized by high glucose transporter type 4 (GLUT4) expression and decreased levels of the natural killer group 2d (NKG2D) and NKp46, resulting in decreased function of killing pathogens making them more prone to apoptosis [12]; and (5) the high blood glucose level of patients with DM also damages the humoral immunity through nonenzymatic glycosylation and several protein covalent compounds, which results in functional defects in CD4^+^ and CD8^+^ T cells [12].

**Figure 2:**
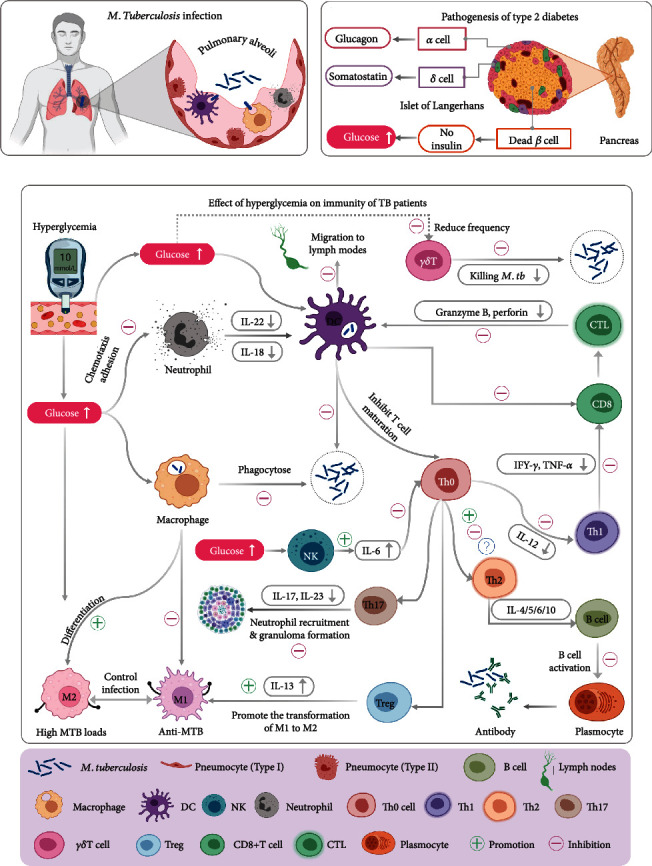
Effects of hyperglycemia on host clearance and killing of *M. tuberculosis*. (a)Schematic diagram of *M. tuberculosis* infection. (b) Pathogenesis of T2DM. (c) Effect of hyperglycemia on the immunity of TB patients.

TB is a respiratory tract transmitted disease. Its pathogen, MTB, is firstly recognized by antigen-presenting cells (APCs) that roam the interstitial and alveolar surfaces, such as alveolar macrophages or DCs (Figure 2(a)). After phagocytosis of MTB, APCs will kill MTB by phagolysosome. Among them, immature dendritic cells (iDCs) will migrate to lymph nodes after capturing MTB. In this process, neutrophils will help iDCs transform into mature dendritic cells (mDCs), activate lymphocytes through antigen presentation, and trigger adaptive immune responses. The native CD4^+^ T lymphocytes (Th0 lymphocytes) will differentiate into Th1 lymphocytes with the help of IL-2 and IL-12 cytokines. The activated Th1 lymphocytes secrete cytokines such as IFN-*γ* and TNF-*α* to enhance the bactericidal ability of macrophages and promote cytotoxic CD8^+^ T cells to secrete granzyme B and perforin to dissolve infected macrophages and eliminate MTB. Interestingly, Th0 lymphocytes also can differentiate into Th2 lymphocytes with the help of IL-4. Th2 T lymphocytes promote the transformation of B lymphocytes to plasma cells by secreting cytokines such as IL-4, IL-5, and IL-10 and induce a humoral immune response. The combined action of innate immunity and adaptive immunity constructs a resistant barrier against MTB infection [13, 14].

As a risk factor for TB, DM can interfere with the host's innate and adaptive immune response to reduce the killing of MTB (Figures 2(b) and 2(c)). Firstly, hyperglycemia can induce the increase of M2 polarization of macrophages, which weakens the phagocytic ability of macrophages and leads to a high load of MTB *in vivo*. Secondly, although hyperglycemia causes an increase in absolute neutrophil count, its phagocytic function decreases [15]. Thirdly, the increased frequency of NK cells secreting TNF-*α*, IL-2, and IL-17F also causes an increase in the severity of the disease or higher bacterial loads [16]. Fourthly, under the influence of DM, the secretion level of DC decreased, which reduces the stimulation of CD4^+^ T cells and reduces the adaptive immune response [17]. Finally, hyperglycemia inhibits the secretion of cytokines such as IFN-*γ* and TNF-*α* by Th1 T lymphocytes and reduces the clearance and killing effect of Th1 immune response on MTB. At the same time, the decrease of IFN-*γ* can also inhibit the activation of CD8^+^ T lymphocytes by Th1-type lymphocytes, reducing the secretion of bactericidal substances such as granzyme, lysozyme, and IFN-*γ*, which reduces the bactericidal ability of cytotoxic T lymphocytes. In patients with TB-DM, the functions of innate and adaptive immune cells are damaged one after another, which weakens the ability of immune cells to inhibit MTB and increases the susceptibility to TB [18].

## 3. Effect of Hyperglycemia on Innate Immune Cells in Patients with TB

The innate immune cells mainly include macrophages, DCs, NK cells, and neutrophils. They usually roam in alveolar tissue and carry out immune patrol and sentry missions. Once the invasive MTB are found, they can capture MTB through pattern recognition receptors (PRRs) and initiate a series of immune mechanisms to eliminate and kill MTB, such as phagocytosis, autophagy, apoptosis, and activation of inflammatory bodies (Table 1) [19].

### 3.1. Macrophages

Macrophages play an essential role in the early and chronic infection of TB by producing reactive oxygen species, reactive nitrogen, and cytokines and inducing autophagy. Alveolar macrophages were the first sentinels to alert the invasion of MTB. Then, they began to recruit other types of macrophages (such as monocyte-derived macrophages) to reach the infection sites [19]. Microenvironment signals differentiate macrophages. M1 and M2 populations are two extreme phenotypes of the macrophage polarization spectrum (Figure 2(c)) [29, 30]. Classic M1 macrophages have a proinflammatory effect and Th1 immunomodulatory properties, which mediate antibacterial defence [20]. Selectively activated M2 macrophages expressed anti-inflammatory, angiogenic, and Th2 immunomodulatory properties [20]. It is reported that DM causes chronic low-grade inflammation in patients with TB. Lopez-Lopez et al. reported that programmed death-ligand 1 (PD-L1) expression on macrophages was increased, but HLA-DR, IL-12p70, IL-1B, and IL-6 were decreased in diabetic compared to healthy subjects [24]. Similar results were found in patients with TB-DM [24]. After infection with MTB, patients with DM expressed a significantly higher level of PD-L1 than patients without DM. PD-L1 is an inhibitor of T cell activation. A high PD-L1/CD86 ratio leads to a susceptibility of macrophages to MTB and immunosuppression of Th1 response. These data suggest that in patients with TB-DM, hyperglycemia changes the activation state of macrophages, harms antigen presentation and activation of Th1 immune response of macrophages, and indirectly affects the ability of macrophages to eliminate MTB [24].

Recent studies have also confirmed the effect of hyperglycemia on M1 macrophages in animal models. Martinez et al. established a mouse model of chronic TB-DM induced by streptozotocin (STZ) [31]. It was found that alveolar macrophages infected with MTB could delay the induction of innate immunity. The decreased expression of macrophage receptors of collagenous structure (MARCO) and CD14 leads to the damage of the sentinel function of alveolar macrophages, which promotes the susceptibility of diabetic hosts to TB. Therefore, the bactericidal ability of suppressed innate immune cells is reduced, resulting in the decrease of the number of dendritic cells migrating to the lymph nodes. Finally, the initiation of adaptive immune response is delayed, and the number of bacteria in the lungs increases exponentially [31]. Coincidentally, Sinha his colleagues also found that hyperglycemia significantly affected immune cells in a mouse model infected with MTB. It was observed that macrophage recognition was impaired in prediabetes, and IFN response was reduced, resulting in a delay in the initiation of adaptive immunity [32]. These results suggest that we should strengthen the control of blood glucose levels in patients with TB before the onset of diabetes to avoid further deterioration of MTB infection. Interestingly, this study also confirmed that obese mice with normal glucose tolerance were better able to control MTB, which indicates that obesity may be a protective factor for patients with TB-DM. These studies perfectly illustrate hyperglycemia's effect on immune cells in patients with tuberculosis in animal models.

As mentioned above, M1 macrophages play an essential role in controlling TB infection. M2 macrophages are usually associated with the multiplication of MTB at the site of infection, resulting in high bacterial loads [20]. A previous study has reported that T2DM can promote polarization of M2 macrophages, induce inflammation at the site of TB infection, and ultimately cause congenital immune abnormalities [21]. To further investigate the effects of the hyperglycemic environment in the alveoli on human alveolar macrophage (hAM), Vance and colleagues simulated the alveolar microenvironment with hyperglycemic coculture conditions and found that MMP9 and CD169 were significantly upregulated in M2 polarization [33]. This study demonstrated that hyperglycemia reduced the ability of macrophages to clear MTB by phagocyte assay.

On the other hand, in clinical research, a great deal of evidences have found that hypoglycemic agents such as metformin and sulfonylureas can effectively reduce the risk of TB-DM [34–36]. Furthermore, van Doorn et al. have found that the antihyperglycemia effect of Poly (ADP-ribose) polymerase (PARP) inhibitors was related to the regulation of macrophage function [37]. PARP inhibitors can promote the secretion of MIP1*α* and MIPI *β* by M1 macrophages, inhibit the production of IFN-*α*2 by M2 macrophages, and increase the number of M1 and M2 macrophages [37]. Overall, PARP inhibitors are effective in the treatment of patients with TB-DM. Furthermore, studies have found that glibenclamide could reduce M1, promote M2 polarization, reduce the ability of patients with diabetes to kill MTB, and increase their susceptibility to TB.

In thousands of years of war with the human immune system, MTB has evolved an immune escape mechanism capable of coping with macrophage strangulation. MTB survives by preventing macrophages from maturing, acidifying, or fusing with lysosomes. However, it is also possible that MTB can escape into the cytoplasm, switching from cell death mode to increased necrosis, resulting in granulomas that escape the clearance of the host's immunity and reactivate to cause active TB when the external environment is suitable [38].

### 3.2. Neutrophils

Neutrophils are essential nonspecific immune cells, accounting for 50% to 70% of the total number of white blood cells. These cells have several critical effects: chemotaxis, phagocytosis, and bactericidal effect. In the early stages of MTB infection, neutrophils will be quickly recruited to the infected site to assist macrophages in phagocytosing MTB. Animal studies have shown that neutrophils play an important role in the early stages of TB infection.

Neutrophils arrive at the vaccination site 3-4 hours after BCG vaccination in mice and rabbits [39]. However, in diabetic patients infected with MTB, the function of neutrophils killing MTB is often impaired. Tripathi et al. evaluated the role of cytokines IL-22 and type-3 innate lymphoid cells (ILC3) from neutrophils in regulating inflammation and mortality in T2DM mice infected with MTB [22]. The results showed that the level of IL-22 secretion in diabetic mice infected with MTB was significantly lower than that in nondiabetic mice infected with MTB. Interestingly, after treatment with recombinant IL-22, the damage to alveolar epithelial cells was reduced, and the survival rate of mice was prolonged [22]. Therefore, the IL-22 pathway may be a potential target for intervention in patients with TB-DM.

Additionally, it is reported that IL-8 can attract neutrophils to the site of MTB infection [23]. A previous study revealed that the IL-8 level was not significantly different between TB patients and healthy controls; however, under T2DM condition, IL-8 level showed a continuous downward trend from PDM (subjects with pre-DM) to NDM (subjects with newly diagnosed DM) to KDM (subjects with known DM) [23]. These results suggest that the number of neutrophils recruited by IL-8 to the infected site is also reducing progressively from the pre-DM group to the known DM subjects, which partly explains the worst prognosis of patients in the DM group after infection with MTB. In addition to influencing the innate immune response, the secretion of IL-8 by macrophages in DM patients infected with MTB decreases, but the productions of MCP-1 and RANTES increase [24]. This frequent but unhealthy cooperation between cells leads to chronic low inflammation, which causes the suppression of neutrophils. As a result, unhealthy intercellular collaboration hinders the development of adaptive immune response and leads to the deficiency of Th1 response [24].

The decrease of neutrophil recruitment is related not only to itself but also to the surrounding microenvironment. For example, Raposo-García et al. observed an increase in the absolute count of neutrophils in patients with TB-DM, but a decrease in adhesion led to a reduction in phagocytosis [15]. This may also be one of the causes of decreased immunity in TB-DM patients. Therefore, it is not difficult to find that hyperglycemia changes the microenvironment of neutrophil survival, reduces the neutrophil bactericidal ability, and increases the risk of MTB infection in DM patients.

### 3.3. Natural Killer Cell

As the first line of defence to eliminate infected cells and tumor cells, NK cells can produce regulatory cytokines, such as IFN-*γ* and GM-CSF [40]. However, in patients with TB-DM, the number of NK cells increased, which may be related to the inhibition of the expression of CD4^+^ T cells that promoted the proliferation of NK cells [41]. NK cells are special lymphocytes that express CD16 and CD56. It was reported that the expression of CD16 and CD56 was negatively correlated with the expression of CD3 and CD4 [41]. The expression levels of CD16 and CD56 in patients with TB-DM were significantly higher than those in patients with simple TB [41]. After a 12-month follow-up, CD16 and CD56 of TB-DM patients decreased, indicating that NK cells play a certain role in clinical diagnosis and monitoring the effect of treatment in TB-DM patients. In addition, CD11c is also related to the function of NK cells. In a mouse model, Cheekatla et al. found that the interaction between NK cells and CD11c could promote the secretion of IL-6, increase the levels of pro- and anti-inflammatory factors in diabetic mice infected with MTB, and reduce the survival rate of mice [42]. The increase of IL-6 also inhibits the production of human CD4^+^ T cells and reduces the cellular immune response of Th1 and Th17, which indirectly affects the adaptive immunity [42].

### 3.4. Dendritic Cells

DCs express a high density of MHC-II molecules, which is beneficial to contact with antigens and present them to T cells. Therefore, DCs are the most potent APCs known to date and can activate initial T cells to initiate adaptive immune responses. MTB is a facultative intracellular parasite. Clearance and killing of MTB mainly depend on T cell-mediated cellular immunity, and DCs play a bridge role in activating adaptive immune response [43]. In the initial stage of MTB infection, iDCs are highly concentrated in the infected site to capture MTB and migrate to lymph nodes. Subsequently, these iDCs migrate to secondary lymphoid tissue, and they mature gradually during this process. The mDCs in the T cell region of secondary lymphoid tissue will present antigens, activate T cells, and initiate adaptive immune responses [44].

DCs bridge innate immunity and adaptive immunity through antigen presentation. DCs can be divided into myeloid DCs (DC1) and lymphoid DCs (DC2). After maturation, DC1 mainly secretes IL-12, IL-18, IFN-*γ*, and other cytokines, which can induce the differentiation of Th0 cells into Th1 cells and mediate the cellular immune responses. In contrast, DC2 mainly produces IL-10 and IL-4 cytokines after maturation, which promote the differentiation of Th0 cells into Th2 cells and mediate the humoral immune responses. The previous study has reported that the level of DCs decreased in patients with TB-DM [17]. The total number of DCs and the number of DC1 subpopulations in peripheral blood of TB patients were lower than those of healthy people, but the number of DC2 subpopulations had no significant change [17]. However, the level of DCs in patients with TB-DM was lower before and at the early stage of treatment, and the frequency of DCs was reversed after the completion of treatment. The results showed that the frequencies of DC1 and DC2 were negatively correlated with hyperglycemia load [17]. Therefore, hyperglycemia may be the main factor leading to changes in the frequency of different DC subsets in patients with TB-DM. Hyperglycemia reduced the frequency of both DC subsets, impairing the ability of DCs to activate T cells, thereby attenuating the power of the host to clear and kill MTB.

## 4. Effect of Hyperglycemia on Adaptive Immune Cells in Patients with TB

Innate immune cells play a significantly influential role in the early stage of MTB infection, against invasive MTB and activating T and B lymphocytes to trigger the adaptive immune responses. T lymphocytes activate adjacent immune cells through cytokines, directly or indirectly act on pathogen-infected cells, promote the apoptosis of these cells, and finally eliminate MTB (Table 1). According to the difference in antigen recognition, T cells can be divided into *αβ*T cells (containing *α* and *β* chains) and *γδ*T cells (containing *γ* and *δ* chains). Furthermore, *αβ*T cells can be subdivided into CD4^+^ T cells and CD8^+^ T cells according to their surface characteristic molecules [13, 45, 46].

### 4.1. CD4^+^ T Cells

CD4^+^ T cells are widely regarded as a primary source of IFN-*γ* production and play an essential role in controlling MTB infection. It was found that the response ability of CD4^+^ T cells producing IFN-*γ* and TNF-*α* was negatively correlated with the severity of diabetes. The decrease in the production of IFN and TNF function leads to the deficiency of antigen presentation function, which delays the activation of T lymphocytes by APCs [47]. According to the secretion of cytokines, CD4^+^ T cells can be divided into Th1, Th2, Th17, and regulatory T cell (Treg cell) subsets. Different cell subsets play different roles in the fight against MTB infection. Th1 cells are generally considered the most critical cells against MTB infection. They inhibit the growth and replication of MTB by secreting cytokines such as IFN- *γ*, IL-2, IL-12, and TNF-*α*. Furthermore, they can also activate CD8^+^ T lymphocytes to kill MTB [48]. Th2 cells mainly secrete IL-4 and IL-10 to induce B lymphocytes to transform into plasma cells and mediate humoral immune response [49]. Th17 cells secrete IL-17, induce the expression of chemokine CXCL-13 through the signal of IL-17 receptor (IL-17R) in nonhematopoietic cells, and recruit CXCR5 T cells to form pulmonary lymphoid follicles near the macrophages infected by MTB, thus optimizing the activation of macrophages and the control of MTB [50]. Treg cells express various chemokine receptors such as CCR4, CCR8, and CXCR3, which downregulate the cellular function of Th1 to counteract bad tissue destruction and prevent immunopathology [51].

Keeping the dynamic balance of Th1 and Th2 is the most crucial mechanism for controlling TB infection, and it is also a vital examination index to observe the effect of tuberculosis treatment [52]. Hyperglycemia in patients with DM causes extensive changes in systemic proinflammatory factors and inflammatory inhibitory factors, promotes the secretion of Th2 cytokines, but does not significantly increase the proportion of Th1 cells, resulting in a decrease in the imbalance of Th1 and Th2 and inhibition of antituberculosis immune function [53]. For example, Kumar et al. found that the response of CD4^+^ T cells was increased, the frequencies of Th1 and Th17 were raised, and the frequency of Treg cells was lower in patients with TB-DM [54]. Furthermore, Meenakshi et al. reported that IL-12 has a substantial immunological effect, and the increase in its production can promote the killing of MTB [55]. IL-12 is a potent inducer of IFN-*γ* that can promote MTB elimination *in vivo*. However, the insufficient concentration of reduced glutathione (GSH) leads to inadequate production of IL-12 in patients with TB-DM. The decrease of IL-12 secretion in patients with TB-DM declined the ability of Th1 cells to kill MTB by secreting a high level of IFN-*γ* and increased the susceptibility to TB. In contrast, patients with TB-DM had significantly higher levels of IL-10 than those with TB alone [55]. Increased IL-10 inhibits the Th1 response and exacerbates the severity of TB. Based on the receiver operating characteristic (ROC) analysis, IL-12 is considered the most sensitive and specific potential biomarker to be used to diagnose TB-DM [55]. In discussing the effects of macrophages on TB-DM, the researchers found that PD-L1 increased in expression, which through the PD-1/PD-L1 pathway inhibited Th1 cell proliferation, reduced Th1/Th2 ratio, and ultimately led to a decrease in IFN-*γ* production [24]. Ronacher et al. and Kumar et al. found that Th1 and Th17 cells secrete low proinflammatory cytokines and Th2 cells secrete low anti-inflammatory cytokines in diabetic patients with latent tuberculosis infection (LTBI) [25, 26]. During the transition of LTBI to active TB, both anti-inflammatory and proinflammatory cytokines will increase, and IL-10 will show a higher level [25, 26]. These results suggest that the changes in inflammatory factors reflect the change in MTB loads in patients with TB-DM, and the imbalance between Th1 and Th2 weakens the ability of patients to eliminate MTB [25, 26].

In both mice and humans, there is a group of CD4^+^CD25^+^ Treg cells derived from the thymus, which have an immunosuppressive function, such as high expression of Foxp3, CD45RO, CTLA-4, CCR4, mTGF-*β*, GITR, and CD62L and low expression of CD45RA and CD127 (IL-7R). Treg cells are antagonistic to Th1, Th2, and Th17 cells [45]. Radhakrishnan et al. studied the effect of BCG vaccination on the immune response to MTB infection in mice with T2DM [51]. The results showed that BCG immunization significantly reduced mortality in TB-DM mice, and this benefit was not due to the protective effect of BCG but was related to the increase of IL-13 secreted by CXCR3^+^ Treg in the lungs of TB-DM mice [51]. IL-13 is mainly derived from Treg cells. In TB-DM mice, Treg cells secrete significantly higher levels of IL-13, which indirectly affects the polarization of macrophages and promotes the transformation of macrophages from M1 to M2 [51]. Kumar et al. reported that Treg cells were significantly reduced in patients with TB-DM in the early stages of anti-TB therapy but reversed completely after treatment was completed [56]. Therefore, the Treg cells and their secretion of IL-13 play essential roles in transforming macrophages from M1 to M2 and anti-MTB infection, and the increase of its proportion results in insulin resistance and TB susceptibility [56].

### 4.2. CD8^+^ T Cell

CD8^+^ T cells play a protective role in MTB infection by secreting cytotoxic substances such as granzyme B, perforin, and CD107a to kill the cells infected with MTB [7]. It can also have protective or harmful effects on TB infection by secreting cytokines such as IFN-*γ*, TNF-*α*, IL-4, IL-5, IL-13, IL-17A, and IL-17F [57]. In CD8^+^ T cell subgroups, Tc1 affects the secretion of IFN-*γ*, IL-2, and TNF-*α*; Tc2 affects the secretion of IL-4, IL-5, and IL-13; and Tc17 affects the secretion of IL-17A, respectively [26]. The previous study showed that the frequencies of Tc1, Tc2, and Tc17 cells decreased significantly in DM patients with LTBI, indicating that the function of CD8^+^ T cells was impaired during the latent infection [26]. Kumar et al. further found that although the expression of cytokines secreted by CD8^+^ T decreased when combined with latent infection, the expression of cytotoxic markers representing cytotoxicity increased significantly, and the levels of cytokines and cytotoxic markers were reversed when active TB developed [16, 27]. Wang et al. compared the immune cell profiles between patients with TB complicated with nondiabetes (TB-NDM) and TB-DM. They found that compared to patients with TB-NDM, patients with TB-DM had a higher proportion of Th2 and Th17 cells but a lower proportion of CD8^+^ cytotoxic T cells. There was no difference in the Th1 cell ratio [53]. These data suggest that hyperglycemia inhibits the function of CD4^+^ T cells and CD8^+^ T cells. Taken together, the level and function of CD8^+^ T cells in patients with TB-DM are suppressed from the time of latent infection. In disease development, the reduction of cytotoxic markers reduces the ability to eliminate MTB, confirming the critical role of CD8^+^ T in protective immunity.

### 4.3. *γδ*T Cell

*γδ*T cells are congenital lymphocytes that account for only 1% to 5% of the circulating T lymphocyte and play an important role in triggering the cellular immune responses [58]. *γδ*T cells fight against MTB infection mainly by secreting cytokines, cytotoxic effector molecules, and chemokines. Similar to CD4^+^ T cells, *γδ*T cells can be divided into three subsets: Th1, Th2, and Th17. Kathamuthu et al. explored the role of *γδ*T cells in patients with LTBI-DM, pre-DM (PDM), and non-DM [28]. After MTB antigen stimulation, the frequencies of IFN-*γ*^+^ Th1 and IL-17^+^ Th17 *γδ*T cells were significantly decreased in DM patients with LTBI, indicating that the immune protection of LTBI individuals was impaired. Meanwhile, the cytotoxic molecules such as perforin, granzyme B, and granulolysin secreted by *γδ*T cells decreased significantly in patients with LTBI-PDM and LTBI-DM, resulting in the reduction of the ability to lyse the infected cells [28]. These results suggest that DM, as a risk factor for LTBI to develop into active TB, impairs the immunity of LTBI patients, increases the difficulty of treatment, and leads to poor prognosis [28].

## 5. Current Challenges and Future Opportunities

Although TB is an ancient infectious disease, more and more evidence shows that TB is also an immune-related disease [59]. The confrontation between the invasiveness of MTB and the immunity of the host determines the direction of disease development: (1) if the invasiveness of MTB is stronger than the immunity of the host, it will lead to active TB; (2) if the two forces are equal, LTBI will be observed; (3) if the invasiveness of MTB is weaker than the immunity of the host, MTB will be killed. Coincidentally, the hyperglycemia of patients with DM has a wide and profound effect on the innate immune cells and adaptive immune cells, which makes the immune system impaired. Thus, a high blood glucose environment and low immunity provide ideal conditions for the massive reproduction of MTB.

### 5.1. The Pathogenesis of TB-DM Remains Unknown

A growing number of studies have found that hyperglycemia has weakened the host's immunity, promoting the occurrence and development of TB, and the prognosis of TB treatment is even worse. However, some studies have put forward the contrary view on the change in TB-DM immune mechanism. The main reason for the heterogeneity in the research results is that we do not know enough about the pathogenic mechanism of TB-DM, which makes us lack unified standards in the experimental design. Therefore, in the future, we must strengthen the in-depth study of the pathogenic mechanism of TB-DM, which will deeply explain the impact of hyperglycemia on the host immune system, and lay the foundation for precise treatment.

### 5.2. More Clinical Trials with a Large Sample Size and High-Quality Design Are Needed

In the current research and investigation, it is found that some cytokines such as IL-22 and IL-8 and the ratio of Th1/Th2 have particular monitoring significance for the treatment and diagnosis of TB-DM. In addition, changes in monocyte subsets are also a hallmark of TB-DM. Studies have reported that the secretion of monocyte markers sCD14 and sCD163 in TB-DM is higher than that in TB [60]. sCD14 and sCD163 were positively correlated with bacterial load *in vivo* and were significantly decreased after metformin treatment [60]. Therefore, systemic monocyte markers are clinically used as one of the detection indicators to reflect the severity of the disease and the response to treatment. The new study showed that mean platelet volume (MPV) and plateletcrit (PCT) were significantly decreased in patients with TB-DM, and the sensitivity and specificity of MPV for differentiating TB from TB-DM were 60.8% and 66.4%, respectively [61]. Studies have shown that MPV and PCT are hopeful as good clinical laboratory markers, which can provide some help for the early diagnosis and prevention of TB-DM. Although these indicators have statistical significance in preclinical and small sample size studies, they have not been verified in large-scale clinical trials. Therefore, further confirmation is needed.

### 5.3. The Emergence of New Technologies Provides Potential Tools for Diagnosing and Treating TB-DM

With the progress of scientific research, it has been found that only studying one direction (genome, proteome, transcriptome, etc.) cannot explain all biomedical problems. Scientists propose to study human tissue and cell structure, genes, proteins, and their molecular interactions from an overall perspective and reflect the function and metabolism of human tissues and organs through comprehensive analysis. Omics provides new ideas for exploring the pathogenesis of human diseases. Currently, omics mainly include genomics, proteomics, metabolomics, transcriptomics, lipidomics, immunologics, glycomics, RNomics, radiomics, and ultrasomics. For example, it has been found that there is a significant difference in the expression of the *hspX* gene and *rfpD* gene between patients with TB-DM and DM, which may be a gene target for intervention therapy [62].

Furthermore, some researchers use metabolic syndrome technology to find methionine changes in the development of TB-DM. They observed that methionine is not affected by antibiotic treatment, so it can be used as a detection index to monitor the prognosis of patients [63]. Other researchers have found that changes in intestinal flora play an essential role in patients' immunity, in which short-chain fatty acid: butyric acid is its representative. Butyric acid can inhibit the secretion of IFN- *γ*, IL-22, and IL-17 and promote the secretion of the anti-inflammatory factor IL-10, thus increasing the susceptibility of DM patients to TB. Therefore, it is also beneficial to the treatment of patients by changing the level of intestinal butyric acid [64].

## 6. Summary

TB-DM is a codisease state, and both diseases affect the host's immunity. A large number of studies have shown that DM does increase the risk of TB infection. For the innate immune system, (1) DM affects the polarization of macrophages and reduces the ability of macrophages to present antigens and clear TB; (2) the decrease of cytokines such as IL-22 and IL-8 in TB-DM leads to the decrease of phagocytosis and bactericidal activity of neutrophils; (3) the inhibition of adaptive immunity leads to the increase of NK cells, but its specific effect is not clear; and (4) the decrease of DCs frequency reduces the antigen presentation, resulting in adaptive immune dysfunction. On the other hand, adaptive immunity is the leading force in eliminating MTB infection. DM leads to a decrease in the frequencies of several adaptive immune cells, which in turn leads to a decrease in the level of a variety of cytokines or toxic molecules, resulting in increased survival of MTB *in vivo* and recurrence of active TB.

Furthermore, it is also mentioned that the immune system has changed in the early stages of the development of the two diseases. Therefore, we should follow the treatment principles of early detection, early diagnosis, and accurate treatment, which dramatically reduces the incidence and risk of TB-DM. Therefore, whether it is the research on traditional immunity or the application of emerging technologies, the combination of the two studies can be reasonably applied to clinical diagnosis and treatment. It is also of great significance to reduce the incidence and risk of TB-DM.

## Figures and Tables

**Table 1:** Effect of hyperglycemia on innate and adaptive immune cells in patients with TB.

Immune cells	Mechanism	Expression changes in TB-DM	Major secretory factor	References
Macrophages	Elimination of TB by the production of reactive oxygen species, reactive nitrogen species, cytokines, autophagy, and antigen presentation	(1) Inhibit antigen presentation and Th1 immune response and change the activation state of macrophages (2) Impaired sentinel function decreases alveolar macrophages, resulting in delayed bacterial reproduction and the initiation of adaptive immune responses (3) M1 polarization decreases; M2 polarization increases	M1: TNF-*α*, IL-1, IL-12, type І IFN M2: IL-10	[19–21]
Neutrophils	Rapidly recruited to the site of infection to assist macrophages in engulfing MTB	(1) Decreased neutrophils and the cytokines they secrete (2) Neutrophil influx inhibition hinders the adaptive immune response (3) The change of the neutrophil microenvironment leads to weakening adhesion ability	IL-22, IL-8	[15, 22, 23]
NK cells	Producing immunomodulatory cytokines to clear infected cells from the body	NK cells increase the secretion of IL-6 and inhibit the secretion of CD4^+^ T cells	CD16, CD56	[15]
DC	(1) Antigen presentation to T cells (2) Activation of naive T lymphocytes to initiate adaptive immune responses (3) A bridge between innate immunity and adaptive immunity	(1) DC cells are secreted by diabetes (2) The cytokine secretion frequency of DC subsets changed, the frequency of DC1 decreased, and DC2 did not change significantly	DC1:IL-12, IL-18, and IFN-*γ*; DC2: IL-10 and IL-4	[17]
CD4^+^ T cells	Secretion of cytokines such as IFN-*γ* and TNF-*α* to inhibit MTB growth	(1) CD4^+^ T secretion of cytokines decreased (2) Decrease in the Th1/Th2 ratio and a decrease in IFN-*γ* secretion of Th1 (3) Th17 secretion of cytokines decreased (4) Promote Treg antagonizes with Th1, Th2, and Th17; increases Treg secretion; and promotes M2 polarization of macrophages	Th1: IFN-*γ*, IL-2, IL-12, TNF-*α* Th2: IL-4, IL-10	[24–26]
CD8^+^ T cells	Killing cells infected with MTB by secreting cytokines or cytotoxic substances	(1) The cytokine secretion of CD8^+^ T cells and their subsets decreased (2) During the transformation from LTBI to TB, the secretion of cytotoxic substances decreased gradually, and the secretion of cytokines increased	Granzyme B, perforin, CD107a, IL-4, IL-5, IL-13, IL-17A, IL-17F	[16, 27]
*γδ*T cell	Resist MTB infection by producing cytokines, cytotoxic effector molecules, and chemokines	(1) Decreased cytokine secretion of *γδ*T cells and their subsets (2) Decreased secretion of cytotoxic substances	Th1, Th17, granzyme B, perforin	[28]

## Data Availability

All data generated or analyzed during this study are included in this published article.

## Abbreviations

APC: Antigen-presenting cells
DM: Diabetes mellitus
DC: Dendritic cells
GLUT4: Glucose transporter type 4
GSH: Glutathione
hAM: Human alveolar macrophage
HFD: High-fat diet
IL-17R: IL-17 receptor
IDF: International Diabetes Federation
ILC3: Type-3 innate lymphoid cells
LTBI: Latent tuberculosis infection
MPV: Mean platelet volume
MARCO: Macrophage receptor with collagenous structure
MTB: Mycobacterium tuberculosis
NK: Natural killing cells
NKG2D: The natural killer group 2d
PCT: Plateletcrit
PD-L1: Programmed death-ligand 1
PARP: Poly (ADP-ribose) polymerase
ROC: Receiver-operating-characteristic
ROS: Reactive oxygen species
STZ: Streptozotocin
Treg cell: Regulatory T cells
TB: Tuberculosis
TB-DM: Tuberculosis complicated with diabetes
TB-NDM: Tuberculosis complicated with nondiabetes
T1DM: Type I diabetes
T2DM: Type II diabetes.
